# Identification of the DEAD box RNA helicase DDX3 as a therapeutic target in colorectal cancer

**DOI:** 10.18632/oncotarget.4873

**Published:** 2015-08-01

**Authors:** Marise R. Heerma van Voss, Farhad Vesuna, Kari Trumpi, Justin Brilliant, Cynthia Berlinicke, Wendy de Leng, Onno Kranenburg, Johan G. Offerhaus, Horst Bürger, Elsken van der Wall, Paul J. van Diest, Venu Raman

**Affiliations:** ^1^ Department of Radiology and Radiological Science, Johns Hopkins University, School of Medicine, Baltimore, MD, USA; ^2^ Department of Pathology, University Medical Center Utrecht, Utrecht, The Netherlands; ^3^ Department of Surgery, University Medical Center Utrecht, Utrecht, The Netherlands; ^4^ Wilmer Eye Institute, Johns Hopkins University, School of Medicine, Baltimore, MD, USA; ^5^ Institute of Pathology, Paderborn, Germany; ^6^ Department of Internal Medicine, University Medical Center Utrecht, Utrecht, The Netherlands; ^7^ Department of Oncology, Johns Hopkins University, School of Medicine, Baltimore, MD, USA

**Keywords:** colorectal cancer, DEAD-box RNA helicases, Wnt signaling, small molecule inhibitors, β-catenin

## Abstract

Identifying druggable targets in the Wnt-signaling pathway can optimize colorectal cancer treatment. Recent studies have identified a member of the RNA helicase family DDX3 (*DDX3X*) as a multilevel activator of Wnt signaling in cells without activating mutations in the Wnt-signaling pathway. In this study, we evaluated whether DDX3 plays a role in the constitutively active Wnt pathway that drives colorectal cancer.

We determined DDX3 expression levels in 303 colorectal cancers by immunohistochemistry. 39% of tumors overexpressed DDX3. High cytoplasmic DDX3 expression correlated with nuclear β-catenin expression, a marker of activated Wnt signaling. Functionally, we validated this finding *in vitro* and found that inhibition of DDX3 with siRNA resulted in reduced TCF4-reporter activity and lowered the mRNA expression levels of downstream TCF4-regulated genes. In addition, DDX3 knockdown in colorectal cancer cell lines reduced proliferation and caused a G1 arrest, supporting a potential oncogenic role of DDX3 in colorectal cancer.

RK-33 is a small molecule inhibitor designed to bind to the ATP-binding site of DDX3. Treatment of colorectal cancer cell lines and patient-derived 3D cultures with RK-33 inhibited growth and promoted cell death with IC50 values ranging from 2.5 to 8 μM. The highest RK-33 sensitivity was observed in tumors with wild-type *APC-*status and a mutation in *CTNNB1*.

Based on these results, we conclude that DDX3 has an oncogenic role in colorectal cancer. Inhibition of DDX3 with the small molecule inhibitor RK-33 causes inhibition of Wnt signaling and may therefore be a promising future treatment strategy for a subset of colorectal cancers.

## INTRODUCTION

Although significant advancements have been made in the prevention and treatment of colorectal cancer, this disease still ranks third on the list of causes of cancer related deaths in the United States, which underlines the need for development of new targeted therapies in this field. [1] On a genomic level colorectal cancer is frequently characterized by loss of the tumor suppressor gene p53 and activation of the RAS-RAF signaling pathway, alterations that are common in a multitude of solid tumors. In addition, activation of the Wnt/β-catenin signaling pathway is prevailing and more specific to the colorectal cancer setting, where genetic aberrations in this pathway are found in over 90 percent of cases. The most common alteration is inactivation of the *APC* gene (>70%). Activating mutations in *CTNNB1*, the gene encoding for β-catenin, are less prevalent (5–10%). [2] Thus, identifying druggable targets in this pathway would be beneficial for optimizing colorectal cancer treatment.

Within this context, we identified a member of the RNA helicase gene family, DDX3, which exhibits oncogenic properties in breast and lung carcinomas. [3, 4] In order to therapeutically exploit the benefits of abrogating DDX3 activity in these cancers, we developed a small molecule inhibitor, RK-33, designed to bind to the ATP-binding domain of DDX3 and inhibit its RNA-helicase activity. [4] Potent anti-cancer activity was observed in lung cancer mouse models after DDX3 inhibition by RK-33.

Recent studies indicate that DDX3 is a multilevel activator of the Wnt-signaling pathway. DDX3 was identified as an allosteric activator of CK1ε and hereby promotes phosphorylation of the scaffold protein dishevelled, which activates Wnt signaling during *Caenorhabditis elegans* and *Xenopus* development and in mammalian HEK293t cells. This function of DDX3 was independent of its RNA-helicase activity. [5] In addition, DDX3 was found to regulate the stability of β-catenin protein expression in a helicase-dependent manner through translational regulation of Rac1. [6] In addition, our group identified a direct interaction between DDX3 and β-catenin and its functional role in regulating TCF-4 mediated transcriptional activity in lung cancer cell lines. [4] Notably, DDX3 activity has also been linked to Wnt-signaling activity by the identification of coinciding *CTNNB1* and *DDX3X* activating mutations in Wnt-type medulloblastomas. [7–9]

These mechanistic studies all indicate an important role of DDX3 in Wnt signaling in both normal and transformed cells, but focus on a situation without activating mutations in the Wnt-signaling pathway. It remains to be determined whether colorectal cancer cells, which usually harbor activating mutations in the Wnt-signaling pathway, are dependent on DDX3 as well. In this study, we aimed to evaluate DDX3 as a potential player in the constitutionally activated Wnt signaling that drives colorectal cancer and to assess whether DDX3 inhibition by the small molecule RK-33 is a suitable therapeutic strategy in this cancer type.

## RESULTS

### DDX3 inhibition results in growth inhibition in colorectal cancer cell lines

**Figure 1:**
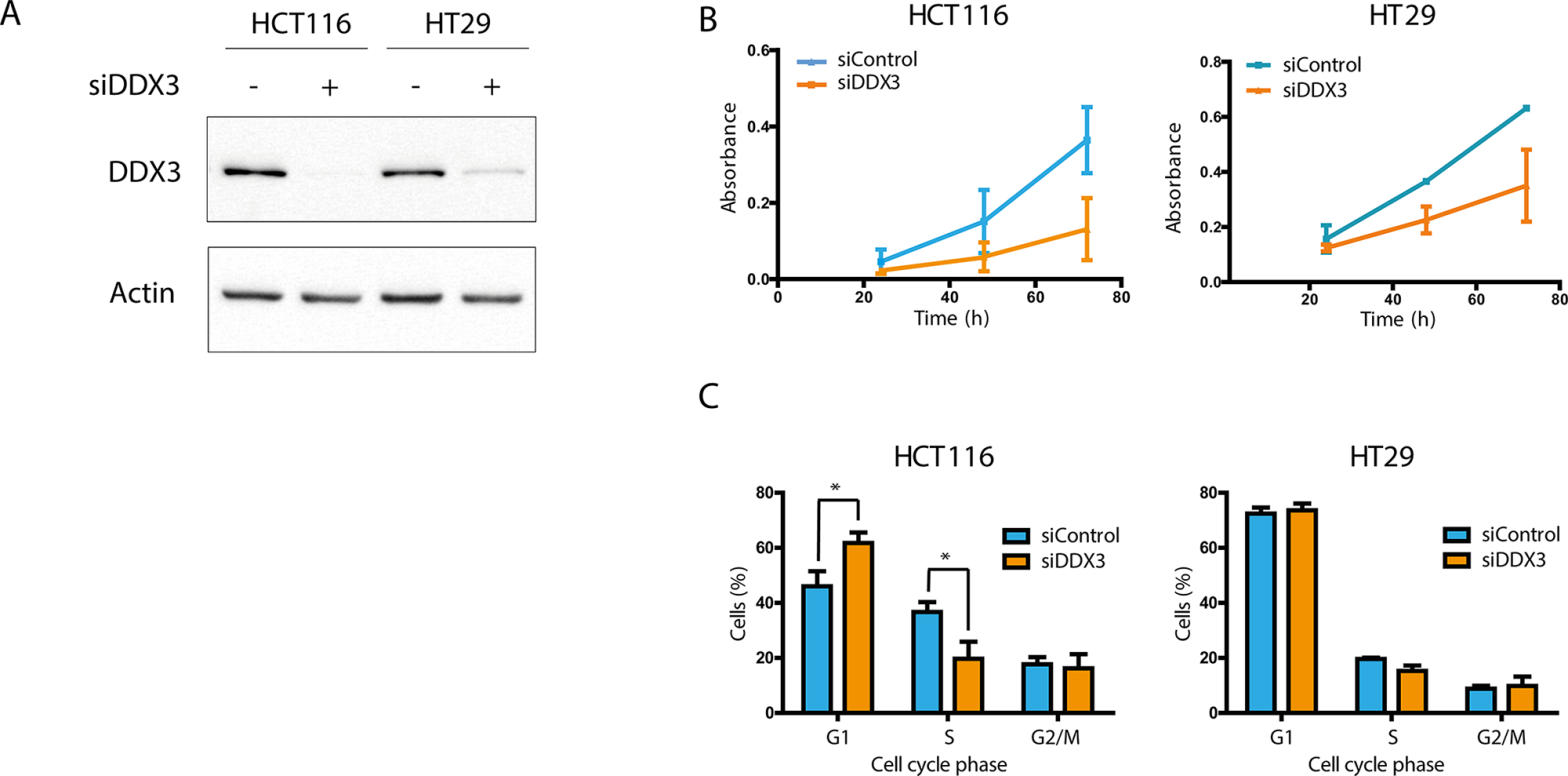
DDX3 dependency in colorectal cancer cell lines **A.** Immunoblots of DDX3 expression in colorectal cancer cell lines HCT116 and HT29 before and after inhibition of DDX3 with 50 nM siDDX3. **B.** Proliferation of Colorectal cancer cell lines after knockdown of DDX3, measured by daily MTS assays. **C.** Cell cycle analysis after knockdown of DDX3. All experiments were performed three independent times, graphs represent mean ± SD, *p < 0.05

To assess DDX3 dependency, we used siRNA to knock down DDX3 expression in the colorectal cell lines HCT116 and HT29 (Figure 1A). DDX3 knockdown resulted in a reduction of cell proliferation in both cell lines (Figure 1B). To evaluate whether the reduction of viable cells was the result of reduced proliferation or increased cell death, we performed cell cycle analysis by flow cytometry on these cell lines after treatment with siDDX3. As seen in Figure 1C, cell cycle analysis indicated a clear G1 arrest in HCT116 cells with a 15.8% increase in G1-phase (*p* = 0.02) and a 17.0% decrease of cells in S-phase (*p* = 0.01). In addition, a slight decrease in S-phase was observed in HT29 (3.4%; *p* = 0.05). These results indicate that these colorectal cell lines are dependent on DDX3 for cell cycle progression.

### DDX3 expression in colorectal cancer patient samples

**Figure 2:**
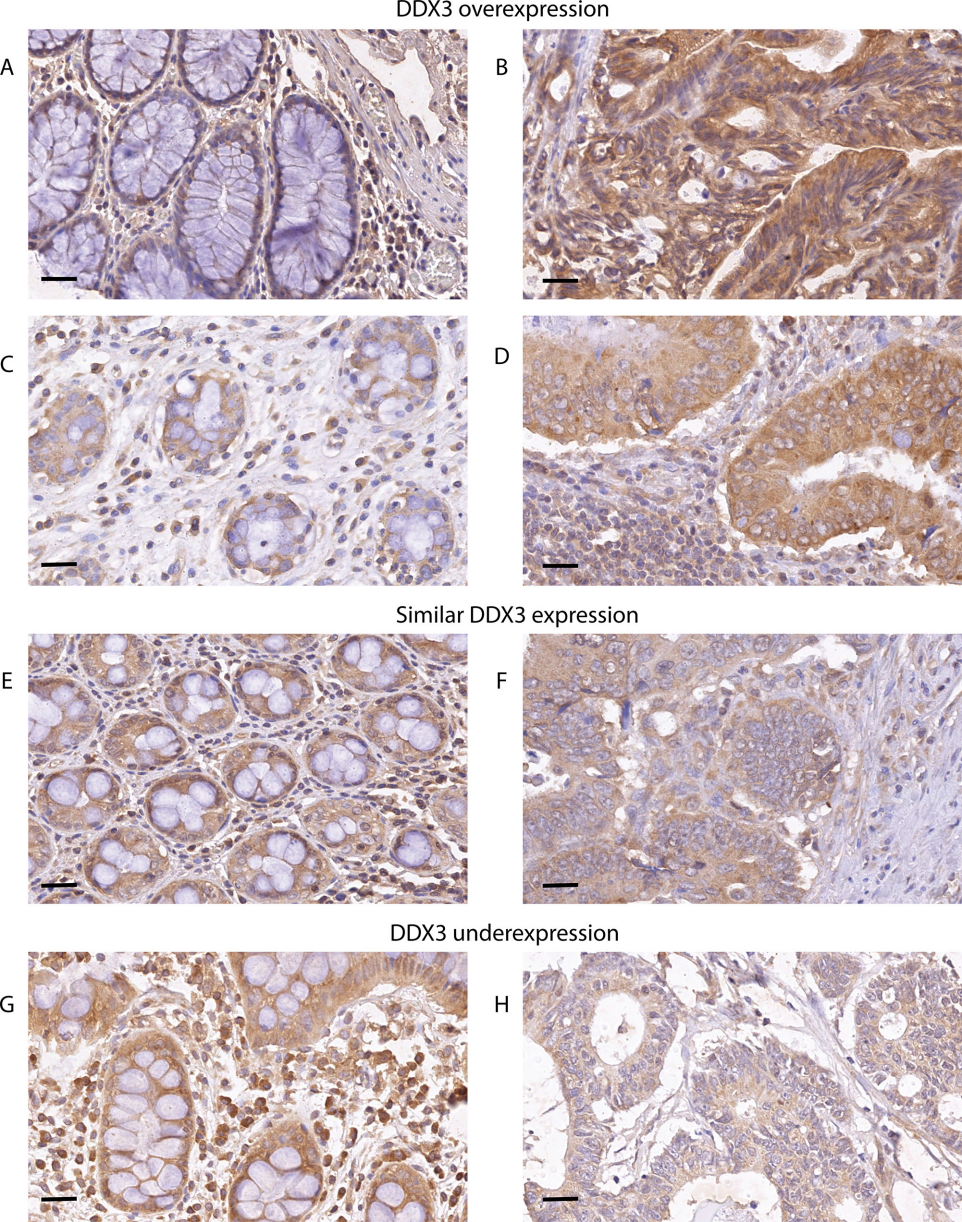
DDX3 is overexpressed in patients with colorectal cancer DDX3 is overexpressed in 39% of patients. Low DDX3 expression in normal colon epithelium **A. and C.**. High DDX3 expression in colorectal adenocarcinoma cells of the same patients **B.** and **D.** 54.2% of patients have similar levels of DDX3 expression in the normal mucosa **E.** and corresponding invasive cancer **F.** Only 6.8% of patients have decreased DDX3 in the invasive tumor **H.** when compared to adjacent normal mucosa **G.** 40 × magnification, scale bar indicates 25 μm

To evaluate whether DDX3 is also expressed in colorectal cancers, we immunohistochemically stained 303 colorectal cancer specimens for DDX3 (Figure 2). High cytoplasmic DDX3 expression was present in 124 samples (40.9%). Corresponding normal mucosa was available for 59 cases. Intratumoral expression was higher in 23 patients (39.0%; Figure 2A–2D), similar in 32 patients (54.2%; Figure 2E–2F) and lower in 4 patients (6.8%; Figure 2G–2H), when compared to the surrounding morphologically normal mucosa.

Next, we compared DDX3 expression to other known clinicopathological characteristics (Table 1). Within this cohort of samples, DDX3 expression did not correlate with any of the other clinicopathological variables.

**Table 1:** Clinicopathological characteristics of DDX3 low and DDX3 high colorectal cancers

		Total		Low DDX3		High DDX3				
		*n*	%	*n*	%	*n*	%	*P*-value	RR	95% CI
**Total**		303	100.0	179	59.1%	124	40.9%			
**Sex**	**Male**	169	55.8%	102	57.0%	67	54.0%	0.61	1.08	0.84–1.39
	**Female**	134	44.2%	77	43.0%	57	46.0%			
**TNM stage**	**1**	22	9.6%	13	9.7%	9	9.4%	0.73		
	**2**	98	42.6%	54	40.3%	44	45.8%			
	**3**	80	34.8%	47	35.1%	33	34.4%			
	**4**	30	13.0%	20	14.9%	10	10.4%			
**Differentiation grade**	**Well**	16	5.3%	8	4.5%	8	6.5%	0.62		
	**Moderate**	228	75.7%	134	75.3%	94	76.4%			
	**Poor**	57	18.9%	36	20.2%	21	17.1%			
**Site of origin**	**Rectum**	95	31.4%	56	31.3%	39	31.5%	0.98	1.00	0.85–1.16
	**Colon**	208	68.6%	123	68.7%	85	68.5%			
**Tumor size**	**<40 mm**	49	22.0%	24	19.0%	25	25.8%	0.44		
	**40–60 mm**	120	53.8%	69	54.8%	51	52.6%			
	**>60 mm**	54	24.2%	33	26.2%	21	21.6%			
**Age at time of diagnosis**	**<65 years**	82	27.1%	54	30.2%	28	22.6%	0.25		
	**65–80 years**	167	55.1%	92	51.4%	75	60.5%			
	**>80 years**	54	17.8%	33	18.4%	21	16.9%			
**Surgical margins**	**negative**	230	99.1%	135	100.0%	95	97.9%	0.17		
	**positive**	2	0.9%	0	0.0%	2	2.1%			

Baseline characteristics of colorectal cancer patients with low and high DDX3 expression. *P*-values are determined by a chi-square test.

### High DDX3 expression correlates with nuclear β-catenin

**Figure 3:**
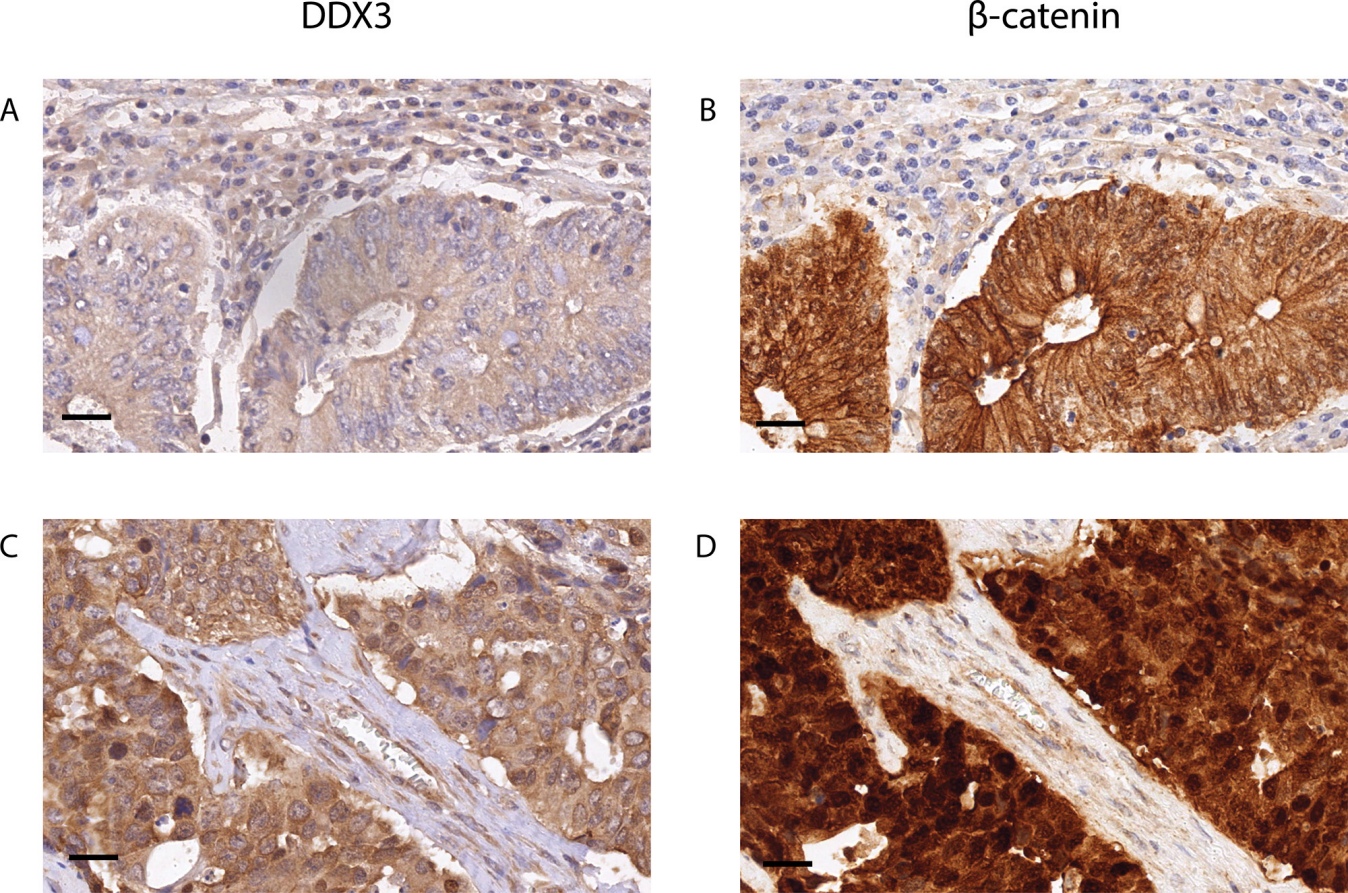
High DDX3 expression is associated with nuclear β-catenin in colorectal cancer samples Low DDX3 expression **A.** is associated with strong expression of β-catenin on the membranes and absence of β-catenin in the nuclei **B.** High DDX3 expression **C.** is associated with increased β-catenin expression in the cytoplasm and the nucleus **D.** 40 × magnification, scale bar indicates 25 μm

Given the activating role of DDX3 in Wnt signaling in other settings, [4] we wanted to determine whether high DDX3 expression is associated with activated Wnt signaling in colorectal cancer patient samples as reflected by an increased cytoplasmic and nuclear β-catenin pool (Figure 3). We separately scored the membranous, cytoplasmic and nuclear localization of β-catenin in tumors with low and high DDX3 expression, which is shown in Table 2. Nuclear β-catenin expression was significantly more prevalent in the DDX3 high group (59.3%) when compared to the DDX3 low group (33.5%; RR = 1.77; 95% CI = 1.36–2.31; *p* = 2.47 × 10^−5^), indicating a connection between DDX3 levels and nuclear β-catenin accumulation. In addition, a trend was observed for more frequent overexpression of cytoplasmic β-catenin in DDX3 high tumors (73% vs 63%; RR = 1.16; 95% CI = 0.99–1.36; *p* = 0.08), which often coincides with nuclear β-catenin expression as shown in Figure 3D.

**Table 2:** β-catenin expression in DDX3 low and DDX3 high colorectal cancers

		Total		Low DDX3		High DDX3				
		*n*	%	*n*	%	*n*	%	*P*-value	RR	95% CI
**Membranous β-catenin**	**complete expression**	231	83.4%	136	83.4%	95	83.3%	0.82		
	**partial expression**	29	10.5%	16	9.8%	13	11.4%			
	**loss of expression**	17	6.1%	11	6.7%	6	5.3%			
**Cytoplasmic β-catenin**	**normal expression**	91	32.9%	60	37.0%	31	27.0%	0.08	1.16	0.99–1.36
	**overexpression**	186	67.1%	102	63.0%	84	73.0%			
**Nuclear β-catenin**	**<10%**	153	55.8%	107	66.5%	46	40.7%	2.47 × 10^−5^	1.77	1.36–2.31
	**>10%**	121	44.2%	54	33.5%	67	59.3%			

Subcellular localization of β-catenin in colorectal cancer samples with low and high DDX3. *P*-values calculated by a chi-square test.

### Sensitivity of colorectal cancers to RK-33, a small molecule inhibitor of DDX3

**Figure 4:**
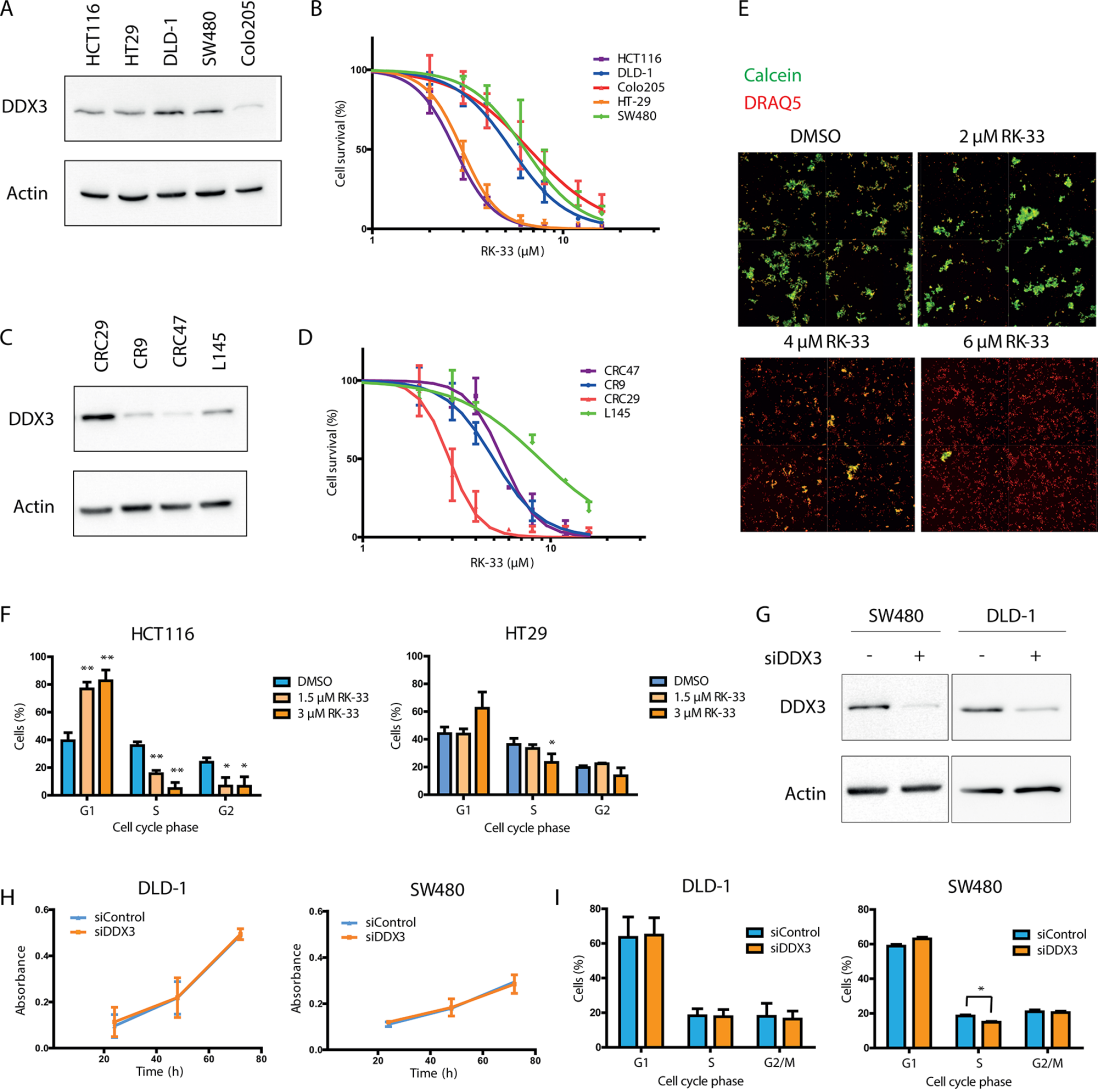
RK-33 sensitivity in colorectal cancer cell lines **A.** Immunoblot showing the relative DDX3 expression in adherent colorectal cancer cell lines. **B.** MTS assay showing cytoxicity of RK-33 in different colorectal cancer cell lines. **C.** Immunoblot showing the relative DDX3 expression in patient-derived 3D cultures. **D.** Cytotoxicity assay showing the sensitivity of patient-derived 3D cultures of colorectal cancer. **E.** Example of cytotoxicity assay with RK-33 in CRC29 3D cultures. The DRAQ5 positive (red) areas are used to determine the outline of the spheroids. The Calcein AM (green) intensity within this area is used as a measure for living cells. **F.** Cell cycle analysis after DDX3 inhibition with increasing concentrations RK-33 in HCT116 and HT29 **G.** Immunoblots of DDX3 expression in colorectal cancer cell lines SW480 and DLD-1 before and after inhibition of DDX3 with 50 nM siDDX3. **H.** Proliferation of Colorectal cancer cell lines SW480 and DLD-1 after knockdown of DDX3, measured by daily MTS assays. **I.** Cell cycle analysis after knockdown of DDX3 in SW480 and DLD-1. All experiments were performed three independent times, graphs represent mean ± SD, *p < 0.05, **p < 0.01

Considering the fact that DDX3 is overexpressed in colorectal cancers, we evaluated the *in vitro* sensitivity of colorectal cancer cells to DDX3 inhibition by RK-33, a small molecule inhibitor of DDX3. Five colorectal cancer cell lines (HCT116, HT29, DLD-1, SW480 and Colo205) were treated with RK-33 and cell viability was assessed by an MTS assay (Figure 4A–4B). All cell lines had an IC50 value in the low micromolar range (3–7 μM). To assess whether RK-33 also showed cytotoxicity in 3D cultures, we expanded our panel with four patient-derived colorectal cancer spheroid cell lines. Spheroid viability after RK-33 exposure was evaluated with an arrayscan (Figure 4C–4E). The spheroids displayed comparable sensitivity to RK-33 as the adherent cell lines (IC50 value range 3–9 μM). Treatment with RK-33 resulted in a G1 arrest in a dose-dependent manner in both HCT116 and HT29 (Figure 4F). An increase in the percentage of apoptotic cells could also be observed in HCT116 after DDX3 inhibition, but not in HT29 (Supplementary Figure 1). The differences in cell cycle distribution were more profound than the increase in apoptotic cells, indicating that the primary effect of DDX3 inhibition is a G1 arrest, which ultimately can result in apoptosis.

Both adherent cell lines and spheroids could be separated into two groups; a sensitive group of cell lines with an IC50 value < 3 μM (HCT116, CRC29, HT29) and a group with a 2–3 fold higher IC50 value ranging from 5–9 μM (CR9, DLD-1, CRC47, SW480, Colo205, L145). Next, we assessed the functional role of DDX3 in the cell lines that were less sensitive to RK-33, by knocking down DDX3 with siDDX3 (Figure 4G). Unlike the RK-33 sensitive cell lines HCT116 and HT29, proliferation in DLD-1 and SW480 was not affected by DDX3 knockdown (Figure 4H) and only a moderate drop in S-phase was observed in SW480 (3.7%; *p* = 0.02). No effect on cell cycle was seen in DLD-1 (Figure 4I).

### Molecular predictors of DDX3 dependency

Centered on our finding of differential sensitivity to RK-33, we hypothesized that sensitivity to RK-33 in colorectal cancer cells may also be associated with other genomic drivers of cellular transformation. Almost all colorectal cell lines and spheroids expressed DDX3 protein (Figure 4A and 4C), but no direct correlation between RK-33 sensitivity and DDX3 expression levels was observed. Next, we assessed the most commonly occurring mutations in our cell line panel by next generation sequencing (Table 3). Interestingly, we found that two of the three RK-33 sensitive cell lines had wild-type *APC* and *TP53*, whereas the less sensitive group had mutations in these genes.

**Table 3:** Correlation between RK-33 sensitivity and mutation status in colorectal cancer cell lines

Cell line	RK-33 Sensitivity		Mutations				MSI status
	IC50 (μM)	95% CI	*APC* Truncation	*TP53*	*RAS/RAF*	*Others*	
HCT116*	2.71	2.64–2.80	−	−	*KRAS*	*PIK3CA, CTNNB1*	MSI
**CRC29**^†^	2.89	2.66–3.14	−	−	*KRAS, BRAF*	*SMAD4*, *STK11*	MSI
HT29*	2.96	2.84–3.08	E853X, T1556fsX3	R273H	*BRAF*	*PIK3CA, SMAD4*	MSS
**CR9**^†^	4.97	4.51–5.48	R1114X, S1503E	R213X, V157A	−	*SMAD4, FBXW7, ERBB2*	
DLD-1*	5.36	5.15–5.59	I1417X	S241F	*KRAS*		MSI
**CRC47**^†^	5.54	5.07–6.05	R1450X	P75LfsX48	−	*PIK3CA*	MSI
SW480*	6.32	5.60–7.15	Q1338X	R273H, P309S	*KRAS*	*SMAD4*	MSS
Colo205*	6.65	6.00–7.37	T1556fsX3	Y103fsX8	*BRAF*		MSS
**L145**^†^	8.77	7.63–10.08	S1436LfsX34	R110P	*KRAS*		MSS

The genomic background of colorectal cancer derived adherent cell lines and 3D cultures and their relative sensitivity to RK-33. Mutational status was derived from:

* publicly available data in the CanSAR database.

† next-generation sequencing.

^−^no mutation detected.

**Figure 5:**
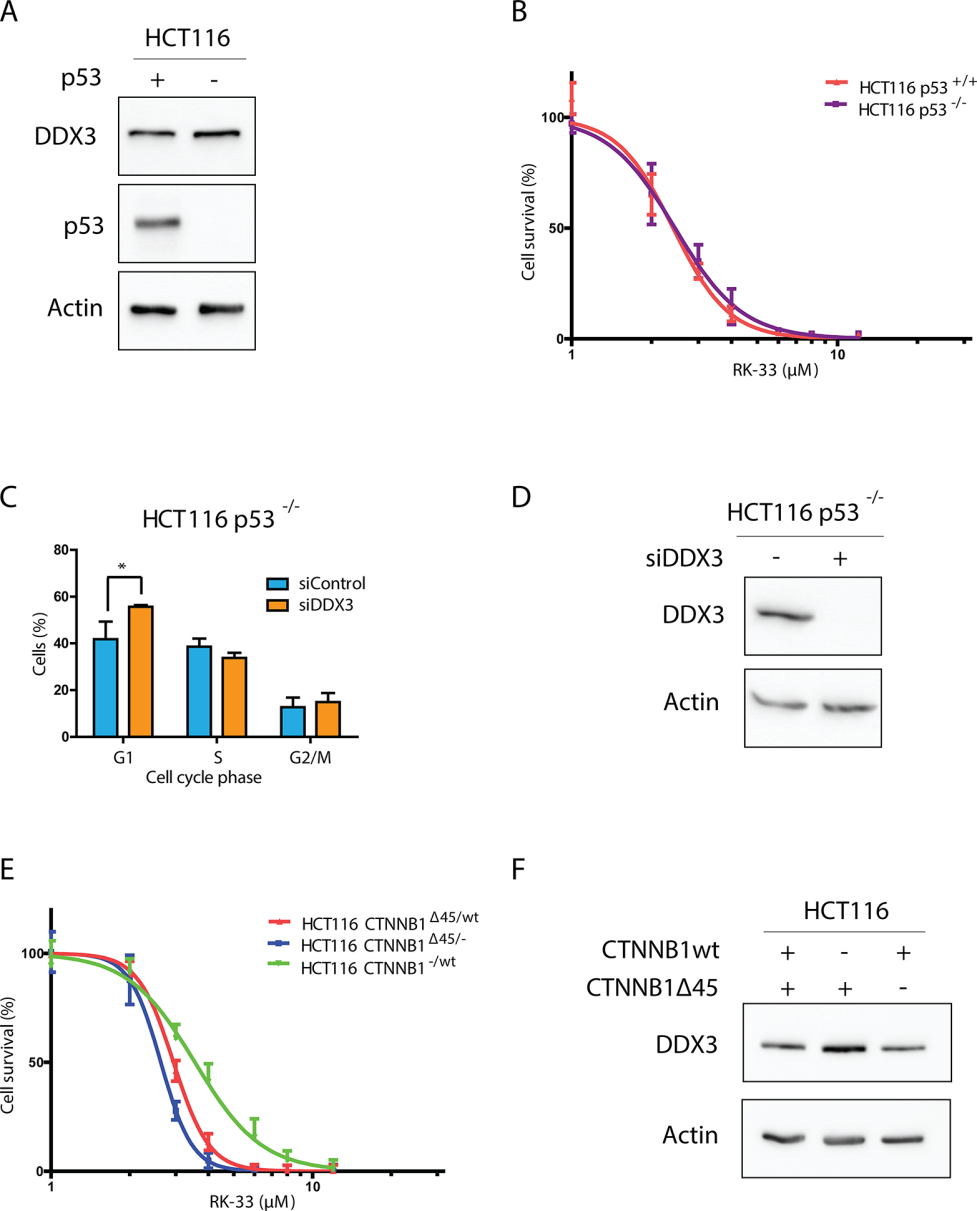
DDX3 dependency in different colorectal cancer genetic subtypes **A.** Immunoblot showing p53 and DDX3 expression in HCT116 with and without p53 **B.** MTS assay showing the relative cytotoxicity after RK-33 treatment in HCT116 with and without p53. **C.** Cell Cycle analysis of HCT116-p53^−/−^ cells after DDX3 knockdown with 50 nM siDDX3 **D.** Immunoblots of DDX3 expression in HCT116 p53^−/−^ before and after inhibition of DDX3 with 50 nM siDDX3. **E.** Relative sensitivity to RK-33 in parental HCT116 (*CTNNB1*^Δ45/wt^) and HCT116 with either the mutant *CTNNB1* allele (*CTNNB1*^−/wt^) or the wild-type allele deleted (*CTNNB1*^Δ45/−^). **F.** Immunoblot showing the DDX3 expression in HCT116 with different β-catenin variants. All experiments were performed three independent times, graphs represent mean ± SD, *p < 0.05

To further determine if *TP53* mutations in cell lines alters sensitivity to RK-33, we compared RK-33 sensitivity in isogenic cell lines with wild-type *TP53* (HCT116-p53^+/+^) and without *TP53* (HCT116-p53^−/−^; Figure 5A). Both cell lines were equally sensitive to DDX3 inhibition with RK-33 (IC50 2.52 *vs* 2.58 μM; Figure 5B). In addition, similar to the parental cell line that expresses p53 (Figure 1C), knockdown of DDX3 resulted in a G1 arrest (13.9% increase; *p* = 0.04) and a decrease of cells in S-phase (4.8%; *p* = 0.11; Figure 5C–5D). This indicates that within our experimental setting the sensitivity of RK-33 is independent of p53 status.

### RK-33 sensitivity in relation to different mutations in the Wnt-signaling pathway

Although DDX3 is thought to play a role in Wnt signaling, cells that harbor an *APC* mutation were less sensitive to RK-33 than cells with wild-type *APC.* Since *CTNNB1* and *DDX3X* mutations co-occur in Wnt-type medulloblastomas [7–9], we hypothesized that DDX3 dependency may be higher in cells with other genetic aberrations in the Wnt-signaling pathway, like mutations in the gene encoding β-catenin. We used HCT116 cells with either the wild-type allele deleted (HCT116 *CTNNB1*^Δ45/−^) or the mutant β-catenin allele deleted (HCT116 *CTNNB1*^−/wt^) to study the contribution of each allele to RK-33 sensitivity. Interestingly, we found that cells with mutant β-catenin were slightly more sensitive (IC50 2.68 μM) than those with only a wild-type allele (IC50 3.71 μM) and that DDX3 expression is slightly higher in HCT116 β-catenin^Δ45/−^ cells (Figure 5D–5E). These results indicate that *APC* wild-type colorectal cancers harboring an activating *CTNNB1* mutation may be more sensitive to RK-33 treatment.

### Inhibition of DDX3 results in reduced Wnt signaling

**Figure 6:**
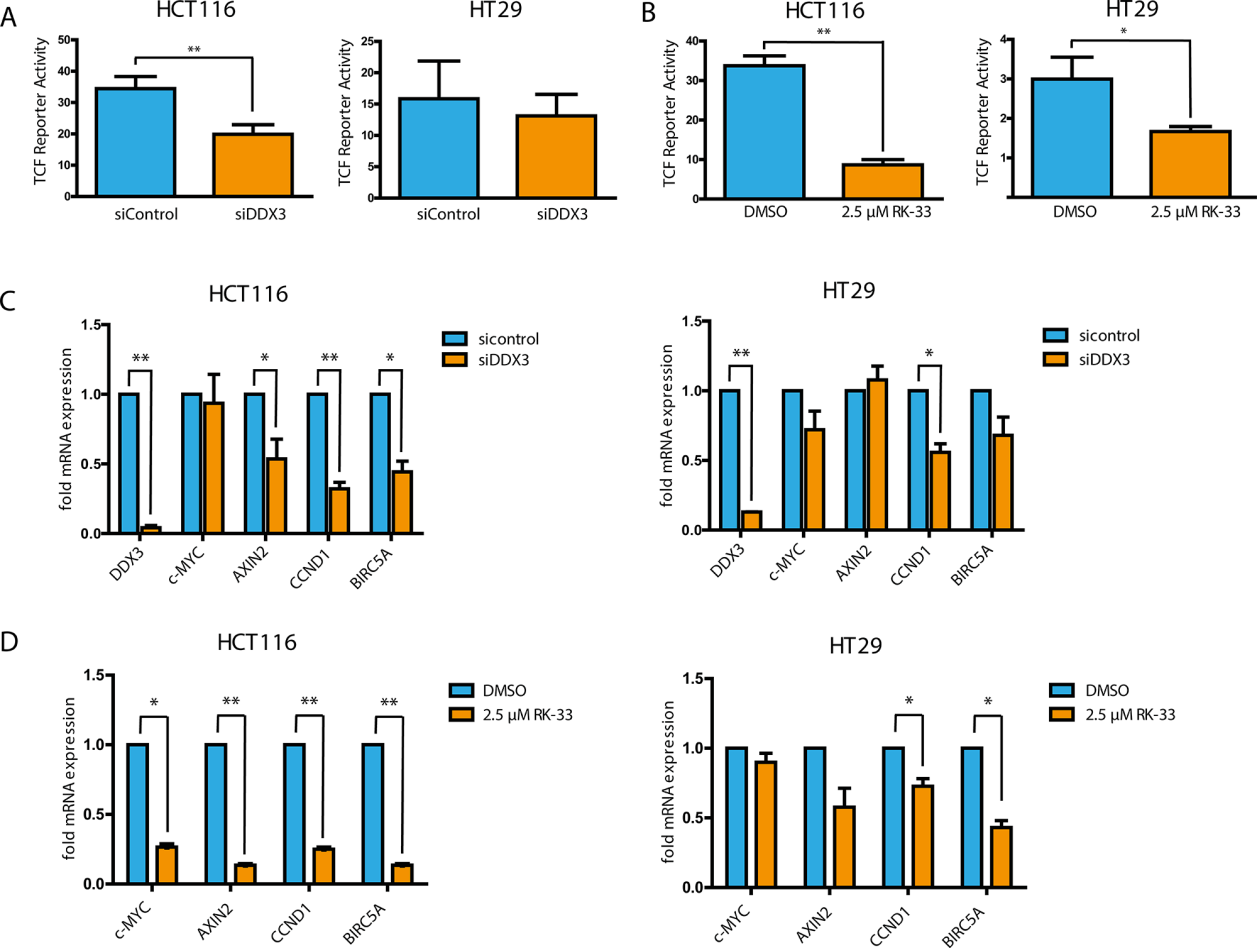
DDX3 inhibition results in reduced Wnt signaling activity **A** and **B.** TCF4-reporter assays after knockdown of DDX3 with 50 nM siDDX3 (A) and inhibition of DDX3 with RK-33 (B) in DDX3-dependent colorectal cancer cell lines HCT116 and HT29. **C** and **D.** Relative mRNA expression of TCF4-target genes after knockdown of DDX3 with 50 nM siDDX3 (C) or DDX3 inhibition with RK-33 (D). All experiments were performed three independent times, graphs represent mean ± SD, *p < 0.05, **p < 0.01.

To evaluate whether the observed proliferation inhibition is the result of interference with oncogenic Wnt signaling, we tested whether DDX3 inhibition causes a reduction in TCF4-promoter activity with a reporter assay. Knockdown of DDX3 resulted in a significant decrease in Wnt signaling in HCT116 (42%, *p* = 0.001) and a small decrease in HT29 (17%, *p* = 0.23; Figure 6A). RK-33 treatment resulted in an even greater inhibition of TCF4-reporter activity of 74% in HCT116 (*p* = 0.0008) and of 44% in HT29 (*p* = 0.03; Figure 6B). To validate whether the reduced TCF4-reporter activity also resulted in reduced mRNA expression of TCF4-regulated genes, we quantified transcript expression for c*-MYC*, *AXIN2*, *CCND1* and *BIRC5A*. As seen in Figure 6C and Supplementary Table 1, DDX3 knockdown resulted in reduced expression of *AXIN2*, *CCND1* and *BIRC5A* in HCT116. Similarly, a decrease was observed in *CCND1*, c*-MYC* and *BIRC5A* expression in HT29. RK-33 treatment also significantly reduced the amount of transcripts of c*-MYC*, *AXIN2*, *CCND1* and *BIRC5A* in HCT116 (Figure 6D). Again this result was slightly less profound in HT29, where RK-33 caused a reduction in *AXIN2*, *CCND1* and *BIRC5A.* Overall, inhibition of DDX3 results in decreased Wnt signaling in HCT116 and to a lesser extent in HT29, which corresponds to their relative dependence on DDX3 for cell cycle progression.

### RK-33 treatment reduces DDX5 protein levels

**Figure 7:**
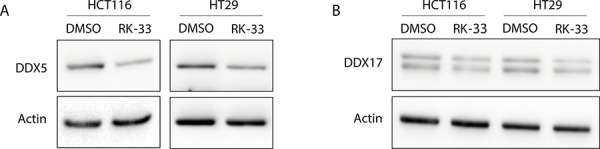
DDX5 and DDX17 expression after treatment with RK-33 **A.** DDX5 expression before and after DDX3 inhibition with RK-33. **B.** DDX17 expression before and after DDX3 inhibition with RK-33.

Since DDX5 and DDX17 are known mediators of Wnt signaling in colorectal cancer [10, 11] and DDX3 and DDX5 have been found to interact [12], we evaluated whether RK-33 treatment also influences DDX5 and DDX17 protein levels. Although we found earlier that RK-33 does not bind directly to DDX5 [4], exposure to RK-33 resulted in decreased DDX5 protein levels, but did not affect DDX17 expression (Figure 7). This indicates that the observed reduction in Wnt signaling could be either as a direct result of decreased DDX3 levels, of the consequentially lowered DDX5 expression, or of a combination of both mechanisms.

## DISCUSSION

Previously, we demonstrated that DDX3 is overexpressed in breast and lung cancers and that targeting DDX3 by RK-33 promotes cell death. [3, 4] This requirement for DDX3 can in part be explained by its involvement in Wnt signaling, as was shown previously by our group and others. [4–6] As the majority of colorectal cancers is driven by mutations in the Wnt-signaling pathway, we explored the possible contribution of DDX3 to Wnt-associated colorectal cancer oncogenesis. In the present study, we showed that DDX3 is overexpressed in 39% of colorectal cancers and that inhibition of DDX3 results in reduced Wnt signaling and a G1 arrest, making DDX3 an attractive therapeutic target in these cancers.

The clinical relevance of the development of Wnt signaling inhibitors which work in a constitutively activated setting is tremendous, since mutations in the Wnt-signaling pathway are not only the first genetic alterations in the adenoma-carcinoma sequence, but advanced colorectal cancers with mutations in *APC* or *CTNNB1* remain dependent on upstream Wnt signaling activity. [13, 14] Especially colorectal cancer stem cells rely on Wnt signaling, and potent inhibition may therefore specifically inhibit the resistant tumor initiating cell population. [15] This potential is also reflected by the cytotoxic effect of RK-33 on 3D cultures of colorectal cancer stem cells in our study. Colorectal cancer drug development is currently limited by a lack of pathway specific targets, potential redundancy of pathway components and toxicity. [16] In this study we show that DDX3 is an integral component of Wnt signaling, and targeting DDX3 by RK-33 is a potential therapeutic option. Although previous mouse studies showed no RK-33-related toxicity [4], future studies will need to validate the anti-cancer activity of DDX3 inhibition in *in vivo* models of colorectal cancer.

Apart from several studies finding an oncogenic role of DDX3 in cancer [3, 4, 6, 17–22], others have suggested that DDX3 may also have a tumor suppressive role in certain cancers. [23–26] Although it is difficult to explain these discrepancies by the molecular backgrounds of different cancer types, it is possible that DDX3 levels can differ between different subsets of patients within a particular cancer. For example, we found high DDX3 expression to be associated with worse prognosis in smoking patients with head and neck squamous cell carcinomas, [27] whereas the opposite was observed in non-smoking patients. [28] DDX3 inhibition resulted in a reduction of proliferation in several colorectal cell lines and all of the colorectal cancer cell lines used in this study were susceptible to RK-33, pleading for reliance of colorectal cancer cells on DDX3 for their survival and arguing against a tumor suppressive role in this particular setting. Interestingly, within our cohort of colorectal cancer cell lines, we observed differential sensitivity to RK-33, indicating that other genetic factors may contribute to oncogenic addiction to DDX3 in neoplastic cells.

Personalized cancer therapy, in which the treatment is adjusted to specific molecular characteristics of a tumor, is of great promise for future cancer treatment. The sensitivity to DDX3 inhibition with both RK-33 and siDDX3 was greatest in HCT116, closely followed by HT29. SW480 and DLD-1 were less sensitive to RK-33 and not affected by siDDX3 treatment. Within our experimental setting, DDX3 dependency was not necessarily reflected by absolute DDX3 protein expression levels. This could be due to the fact that the levels of DDX3 essential to maintain cellular homeostasis are variable in different colorectal cancer cells. Another possibility is that in conjunction with DDX3, there could be an association with other specific genetic alterations that promotes RK-33 sensitivity. DDX3 dependency seemed to be greater in the presence of wild-type APC-status and activating mutations in *CTNNB1*. This finding is in line with the co-occurrence of *DDX3X* and *CTNNB1* mutations in medulloblastomas [7–9] and provides a potential explanation for the fact that HT29, which harbors a mutation in *APC*, does not show a clear G1 arrest upon DDX3 knockdown. However, both our sample size and the differences in RK-33 sensitivity were too small to be able to make any definite claims with regard to predictors of DDX3 dependency.

Different levels of interference have been suggested for DDX3 in the Wnt-signaling pathway. In contrast with our findings, Cruciat, *et al*. found that DDX3 inhibition had no effect on Wnt signaling activity after induction with β-catenin overexpression and that the involvement of DDX3 in this pathway was independent of its helicase activity. [5] It is possible that other mechanisms by which DDX3 is involved in Wnt signaling, like stabilization of β-catenin indirectly through Rac1-signaling [6] or DDX5, or through a direct interaction with DDX3 [4] are more prominent in colorectal cancers. Unfortunately, only a minority of colorectal cancers (23%) falls into the wild-type APC group. However, mutations in *CTNNB1* are highly prevalent in hepatocellular carcinoma (24%), sarcoma (44%) and testicular cancer(24%) [29], suggesting that these cancers may potentially have increased sensitivity to RK-33. In contrast to Sun *et al*. who found DDX3 to be pro-apoptotic in a p53-wildtype breast cancer cell line and anti-apoptotic in cell lines harboring a p53-mutation [30], we found DDX3 dependency not to differ in the presence or absence of p53.

Overall, we conclude that a subset of colorectal cancers is addicted to DDX3 expression. In these more often *APC*-wild-type cancers, inhibition of DDX3 causes a potent reduction of Wnt signaling and a G1 arrest. DDX3 inhibition with the small molecule inhibitor RK-33 is therefore a promising future treatment strategy in colorectal cancer.

## MATERIALS AND METHODS

### Cell lines

HCT116, HT29, Colo205, SW480 and DLD-1 were a kind gift of Professor Fred Bunz (Johns Hopkins University, Baltimore, MD, USA). The HCT116 p53 [31] and β-catenin [32] knockout cell lines were kindly provided by Professor Bert Vogelstein (Johns Hopkins University). All adherent colorectal cancer cell lines were grown in McCoy's 5A supplemented with 10% fetal bovine serum. All cell lines were routinely tested for mycoplasma contamination by a PCR kit (30–1012K, ATCC, Manassas, VA, USA).

The colosphere cultures CR9, CRC29, CRC47 and L145 were a kind gift from Professor Onno Kranenburg (University Medical Center Utrecht, Utrecht, The Netherlands). These cell lines were established from tumor specimens of primary colorectal cancers (CR9, CRC29, CRC47) and colorectal cancer liver metastases (L145). A detailed description of how these colospheres were isolated and maintained has been provided previously. [33] These specimens were obtained in accordance with the ethical committee on human experimentation and informed consent was obtained from all patients. [33] Next generation sequencing was performed to assess the mutation status of 50 commonly mutated genes in these spheroids using the AmpliSeq Cancer Hotpot Panel v2 (LifeTechnologies, Carlsbad, CA, USA) on an Ion PGM platform. Publicly available mutation data of the adherent colorectal cancer cell lines were accessed through the canSAR platform. [34] Clinical information of these cell lines is summarized in Supplementary Table 2.

DDX3 knockdown cell lines were generated by transfecting cells with jetPrime transfection reagent (Polyplus, New York, NY, USA) and 50 nM sicontrol (non-targeting pool) or siDDX3 sequences (ON-*TARGETplus*, Dharmacon, Lafayette, CO, USA).

### Immunoblotting

All cells were harvested at 50–70% confluency. For DDX3 knockdown experiments cells were harvested 72 hours after transfection. For RK-33 experiments cells were harvested after 24 hours exposure to the drug or vehicle control. For whole cellular protein extracts cells were lysed in SDS-extraction buffer (100nM Tris-HCl, 2% SDS, 12% glycerol, 10mM EDTA, pH6.7) and sonicated on ice. 30 μg protein was loaded on 10% SDS-PAGE gels. After gel-electrophoresis proteins were transferred onto PVDF membranes, blocked with 5% milk and probed overnight with primary antibodies against DDX3 (1:1000, mAb AO196) [35], Actin (1:10000, A5441, Sigma-Aldrich, St Louis, MO, USA), DDX5 (1:1000, pab204, EMD Millipore, Billarica, MA, USA), DDX17 (1:1000, Bethyl, Montgomery, TX, USA) and p53 (1:1000, DO-1, Santa-Cruz Biotechnology, Dallas, TX, USA) and followed by appropriate secondary antibodies. The blots were developed with clarity western ECL (Bio-Rad, Hercules, CA, USA) and imaged with G:BOX Chemi XR5 (Syngene, Frederick, MD, USA).

### Proliferation assay and cytotoxicity assays

For the proliferation assays 4–10 × 10^4^ cells were plated in a 24-well plate. The following day the cells were transfected with siDDX3 or sicontrol as described earlier. 48 hours after transfection 2–5 × 10^3^ cells were plated per well in a 96-well plate. The amount of viable cells per well was estimated every 24 hours by an MTS-assay (CellTiter 96 Aqueous One Solution, Promega, Madison, WI, USA).

For this, the cells were incubated with MTS reagent for 2 hours, after which absorbance was measured at 490 nm with a Victor^3^V plate reader (PerkinElmer, Waltham, MA, USA). For the cytotoxicity assays with adherent colorectal cancer cell lines 2–5 × 10^3^ cells were plated per well in a 96-well plate. The following day RK-33 was added. DMSO was added as a vehicle control. Read out occurred after 72 hours of drug exposure with an MTS assay.

The cytotoxicity assays on colosphere cultures have been described extensively elsewhere. [36] Briefly, 80–100 spheroids were plated per well in a 96-well plate with RK-33 or DMSO. After 72 hours of drug exposure the total cell population was labelled with DRAQ5™ (Abcam, Cambridge, UK) and live cells were labelled with Calcein Green AM (LifeTechnologies, Carlsbad, CA, USA). Fluorescence was measured using a Cellomics Arrayscan VTI HCS Reader (Thermo Fisher Scientific, MA, USA). The percentage of dead cells was calculated by normalizing the levels of intensity to and expressed as a relative percentage of the plate-averaged vehicle treated control.

### Cell cycle analysis

Cell cycle analysis was performed as was described previously. [37] In short, for siDDX3 experiments 5–15 × 10^4^ cells were plated per well in a 6-well plate. The following day cells were transfected with sicontrol or siDDX3 and incubated for 72 hours. For experiments with RK-33 4–7.5 × 10^5^ cells were plated in a 6-well plate. The following day cells were incubated for 24 hours with RK-33. Subsequently, cells were harvested and fixed in 70% ethanol overnight at −20°C. Fixed cells were incubated in DNA staining solution (5 μg/ml propidium iodide, 0.5mg/ml RNAse A) for 1 hour. Cell cycle acquisition was performed on a FACScan I or FACSCalibur instrument (BD Biosciences, San Jose, CA, USA). Data was analyzed using FlowJo software (Tree Star Inc., Ashland, OR, USA). Statistical significance was assessed with a student's *t*-test.

### TCF-reporter assays

Transcriptional activity of TCF4 was measured using the dual luciferase assay (Promega, Madison, WI, USA) according to the manufacturer's instructions. For this, cells were transfected with 500 ng TOP-FLASH or FOP-FLASH constructs [38] and 50 ng phRL Renilla constructs as transfection controls, using jetPrime transfection reagent (Polyplus, New York, NY, USA). Luminescence was measured using a luminometer (Berthold Sirius, Oak Ridge, TN, USA).

For the experiments with RK-33 3–4 × 10^4^ HCT116 and HT29 cells were plated in a 24-well plate. After 24 hours the cells were transfected with the TOP/FOP constructs. 7 hours after transfection 2.5 μM RK-33 or DMSO was added for 12–24 hours after which the cells were lysed. For the DDX3 knockdown experiments 12.5–15 × 10^3^ HCT116 and HT29 cells were plated in a 24-well plate. In the evening of the following day the cells were transfected with 50 nM siDDX3 as described earlier. The following morning the cells were transfected with the TOP/FOP constructs and incubated for another 48 hours after which the cells were lysed for the luciferase assay. Relative TCF4-promotor activity was calculated by normalizing TOP-FLASH and FOP-FLASH readings for Renilla luciferase readings and dividing normalized TOP-FLASH readings by normalized FOP-FLASH readings. Statistical significance was evaluated by a paired *t*-test.

### Quantitative reverse transcriptase polymerase chain reaction

HCT116 and HT29 cells were harvested after 12–24 hour exposure to RK-33 or 72 hours after transfection with 50 nM siDDX3. RNA was extracted with an RNeasy kit (Qiagen, Valencia, CA, USA) and cDNA was manufactured using an iScript cDNA synthesis kit (Bio-Rad, Hercules, CA, USA), followed by qPCR using SYBR green (Bio-Rad, Hercules, CA, USA) on an CFX96 Real-Time PCR detection System (Bio-Rad, Hercules, CA, USA). Amplification of 36B4, a housekeeping gene, was used for normalizing gene expression values. Primer sequences: *DDX3* F 5′-GGAGGAAGTACAGCCAGCAAAG-3′, *DDX3* R 5′-CTGCCAATGCCATCGTAATCACTC-3′, *AXIN2* F 5′-TCAAGTGCAAACTTTCGCCAACC-3′, *AXIN2* R 5′-TAGCCAGAACCTATGTGATAAGG-3′, c-*MYC* F 5′-CGTCTCCACACATCAGCACAA-3′, c-*MYC* R 5′-CACTGTCCAACTTGACCCTCTTG-3′, *CCND1* F 5′-GGCGGAGGAGAACAAACAGA-3′ *CCND1* R 5′-TGGCACAGAGGGCAACGA-3′, *BIRC5A* F 5′-CCACCGCATCTCTACATTCA-3′, *BIRC5A* R 5′-TATGTTCCTCTATGGGGTCG-3′. Fold changes in mRNA expression were calculated using the 2^−ΔΔCT^ method. Statistical significance was calculated by performing a paired student's *t*-test on the ΔCT values. [39]

### Patient samples

A tissue microarray (TMA's) with samples from 72 colorectal cancer patients from the Academic Medical Center, Amsterdam was kindly provided by professor Johan Offerhaus (University Medical Center Utrecht). This TMA also included one punch of surrounding normal mucosa per patient. The construction of this TMA has been reported in detail elsewhere. [40] An additional TMA with 292 colorectal cancer samples from Paderborn, Germany was provided by prof. Horst Bürger.

As we used archival leftover pathology material and our study does not affect the included patients, no ethical approval is required according to Dutch legislation. [41] Anonymous or coded use of redundant tissue for research purposes is part of the standard treatment agreement with patients in our hospitals. [42]

### Immunohistochemistry

4 μm sections were cut, mounted on SuperFrost slides (Menzel&Glaeser, Brunswick, Germany), deparaffinized in xylene and rehydrated in decreasing ethanol dilutions. For DDX3 staining, endogenous peroxidase activity was blocked with 1.5% hydrogen peroxide buffer for 15 minutes and was followed by antigen retrieval by boiling for 20 minutes in 10 mM citrate buffer (pH 6.0). Slides were subsequently incubated in a humidified chamber for 1 hour with anti-DDX3 (1:1000, pAb r647). [35] After washing with PBS, slides were incubated with poly-HRP-anti-mouse/rabbit/rat IgG (Brightvision, Immunologic, Duiven, The Netherlands) as a secondary antibody for 30 minutes at room temperature. Peroxidase activity was developed with diaminobenzidine and hydrogen peroxide substrate solution for 10 minutes. The slides were lightly counterstained with haematoxylin and mounted. Positive controls (tonsil) were used throughout. Negative controls were obtained by omission of the primary antibodies from the staining procedure.

β-catenin staining was performed automatically with the Leica BOND RX (Leica Microsystems, Rijswijk, The Netherlands). Antigens were retrieved with Epitope Retrieval Solution 2. The primary antibody against β-catenin (clone 17C2, Novocastra, Eindhoven, The Netherlands) was used in a 1:20 concentration.

Scoring was performed by consensus of two observers (M.H.v.V., P.v.D.). Intensity of cytoplasmic DDX3 expression was scored semi-quantitatively as being absent, low, moderate or strong. The TMAs included multiple cores per patient; the highest score was used for further analysis. Cases with absent to moderate scores were classified as having low DDX3 expression and evaluated against cases with strong expression. Intratumoral DDX3 expression was compared to that of the surrounding normal tissue in case the latter was available for comparison.

β-catenin expression was scored separately for each subcellular compartment. Membrane expression was scored as complete, partial or lost. Cytoplasmic expression was scored as normal or overexpressed. The percentage of positive nuclei was scored. A cut-off of lower or higher than 10% was used for analysis.

Clinicopathological characteristics were compared between DDX3 low and high expressing tumors. Discrete variables were compared by χ2 or Fisher's exact test and odds ratios (OR) were calculated with 95% confidence intervals (95% CI). Statistical analyses were performed using SPSS version 20.0.

## SUPPLEMENTARY FIGURE AND TABLES


