# Supplementary material for: Identification of the DEAD box RNA helicase DDX3 as a therapeutic target in colorectal cancer
Source: Oncotarget. 2015 Aug 1;6(29):28312–26. doi: 10.18632/oncotarget.4873 (PMC4695062; doi:10.18632/oncotarget.4873)
Supplement: Supplementary file 1 [file oncotarget-06-28312-s001.pdf]

SUPPLEMENTARY FIGURE AND TABLES

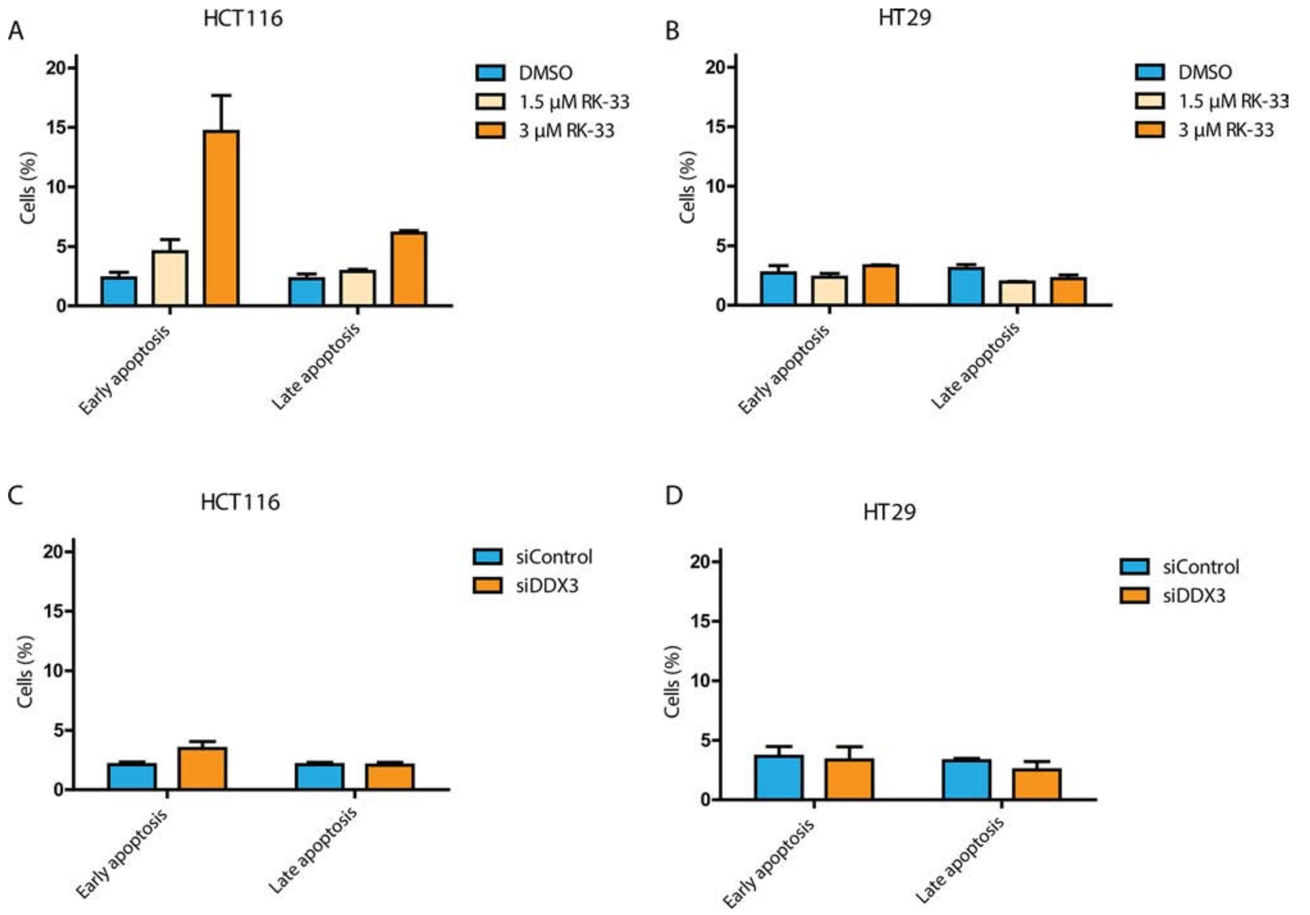

Supplementary Figure S1: Cells undergoing apoptosis after DDX3 inhibition. Histograms depicting the percentage of cells undergoing early apoptosis (Annexin V positive) or late apoptosis (Propidium Iodide labeled) as a result of DDX3 inhibition by RK-33 **A & B.** or siDDX3 **C & D.**, analyzed by flow cytometry. Graph represents mean ± SD.

Supplementary Table S1: mRNA expression of TCF4-target genes after DDX3 inhibition.

		DDX3		*c-MYC*		*AXIN2*		*CCND1*		*BIRC5A*	
		**fold change**	***p*-value**	**fold change**	***p*-value**	**fold change**	***p*-value**	**fold change**	***p*-value**	**fold change**	***p*-value**
**HCT116**	**siDDX3**	23.7	0.005	1.07	0.58	1.86	0.05	3.10	0.005	2.27	0.01
	**RK-33**			3.76	0.01	7.37	0.01	4.68	0.01	7.41	0.001
**HT29**	**siDDX3**	7.81	0.00003	1.40	0.09	1.08	0.30	1.79	0.007	1.47	0.07
	**RK-33**			1.11	0.17	1.73	0.09	1.38	0.03	2.31	0.04

Fold change in mRNA expression of DDX3 and TCF4-regulated genes after DDX3 inhibition with RK-33 or siDDX3. Red = upregulated, green = downregulated. *P*-values calculated by a paired student's *T*-test.

Supplementary Table S2: Cell lines clinical background.

	Patient	Organ	Stage	Derived from	Reference
**HCT116**	48-year old male	Colon ascendens	Dukes' D	Primary tumor	Brattain, et al., [43] Eshleman, et al. [44]
**CRC29**	81-year old woman	Colon	T2N0Mx	Primary tumor	
**HT29**	44-year old female	Colon	Dukes' C	Primary tumor	Fogh, et al. [45]
**CR9**	73-year old male	Colon	T3N0M1	Primary tumor	
**DLD-1**	Male	Colon		Primary tumor	Chen, et al.[46], Dexter, et al. [47]
**CRC47**		Colon (Sigmoid)	T3N1Mx	Primary tumor	
**SW480**	50-year old male	Colon	Dukes' B	Primary tumor	Leibovitz, et al.[48]
**Colo205**	70-year old male	Colon cancer		Ascites	Semple, et al. [49]
**L145**	72-year old male	Colon cancer liver metastasis		Liver metastasis	

Clinical characteristics of the patients from which the cell lines in this study where derived.

SUPPLEMENTARY REFERENCES

1. Brattain MG, Fine WD, Khaled FM, Thompson J, Brattain DE. Heterogeneity of malignant cells from a human colonic carcinoma. Cancer research. 1981; 41:1751–1756.
2. Eshleman JR, Lang EZ, Bowerfind GK, Parsons R, Vogelstein B, Willson JK, Veigl ML, Sedwick WD, Markowitz SD. Increased mutation rate at the hprt locus accompanies microsatellite instability in colon cancer. Oncogene. 1995; 10:33–37.
3. Fogh J. 1975. Human Tumor Cells *in Vitro*. (New York, USA: Plenum Press).
4. Chen TR, Dorotinsky CS, McGuire LJ, Macy ML, Hay RJ. DLD-1 and HCT-15 cell lines derived separately from colorectal carcinomas have totally different chromosome changes but the same genetic origin. Cancer genetics and cytogenetics. 1995; 81:103–108.
5. Gitelman I, Dexter DF, Roder JC. DNA amplification and metastasis of the human melanoma cell line MeWo. Cancer research. 1987; 47:3851–3855.
6. Leibovitz A, Stinson JC, McCombs WB 3rd, McCoy CE, Mazur KC, Mabry ND. Classification of human colorectal adenocarcinoma cell lines. Cancer research. 1976; 36:4562–4569.
7. Semple TU, Quinn LA, Woods LK, Moore GE. Tumor and lymphoid cell lines from a patient with carcinoma of the colon for a cytotoxicity model. Cancer research. 1978; 38:1345–1355.
